# High-frequency conductivity at Larmor-frequency in human brain using moving local window multilayer perceptron neural network

**DOI:** 10.1371/journal.pone.0251417

**Published:** 2021-05-20

**Authors:** Mun Bae Lee, Geon-Ho Jahng, Hyung Joong Kim, Oh-In Kwon

**Affiliations:** 1 Department of Mathematics, Konkuk University, Seoul, Korea; 2 Department of Radiology, Kyung Hee University Hospital at Gangdong, College of Medicine, Kyung Hee University, Seoul, Korea; 3 Department of Biomedical Engineering, Kyung Hee University, Seoul, Korea; University of Queensland, AUSTRALIA

## Abstract

Magnetic resonance electrical properties tomography (MREPT) aims to visualize the internal high-frequency conductivity distribution at Larmor frequency using the B1 transceive phase data. From the magnetic field perturbation by the electrical field associated with the radiofrequency (RF) magnetic field, the high-frequency conductivity and permittivity distributions inside the human brain have been reconstructed based on the Maxwell’s equation. Starting from the Maxwell’s equation, the complex permittivity can be described as a second order elliptic partial differential equation. The established reconstruction algorithms have focused on simplifying and/or regularizing the elliptic partial differential equation to reduce the noise artifact. Using the nonlinear relationship between the Maxwell’s equation, measured magnetic field, and conductivity distribution, we design a deep learning model to visualize the high-frequency conductivity in the brain, directly derived from measured magnetic flux density. The designed moving local window multi-layer perceptron (MLW-MLP) neural network by sliding local window consisting of neighboring voxels around each voxel predicts the high-frequency conductivity distribution in each local window. The designed MLW-MLP uses a family of multiple groups, consisting of the gradients and Laplacian of measured B1 phase data, as the input layer in a local window. The output layer of MLW-MLP returns the conductivity values in each local window. By taking a non-local mean filtering approach in the local window, we reconstruct a noise suppressed conductivity image while maintaining spatial resolution. To verify the proposed method, we used B1 phase datasets acquired from eight human subjects (five subjects for training procedure and three subjects for predicting the conductivity in the brain).

## Introduction

Various techniques to measure and analyze the electrical properties of biological tissues using a magnetic resonance imaging (MRI) scanner have been developed and experimented [1–7]. Using a conventional MRI scanner without any external electrical stimulation, magnetic resonance electrical properties tomography (MREPT) technique successfully recovers the conductivity distribution at Larmor-frequency (about 128 MHz at 3 T) [7–9]. Since the electrical conductivity of biological tissues is primarily determined by the concentration and mobility of ions, a non-invasive and in-vivo high-frequency conductivity imaging has the potential to be sensitive to physiological and pathological conditions of tissues [8]. Using B1 mapping technique with the eddy currents induced by RF pulse, numerous clinical studies using MREPT have been conducted [10–13]. A recent work using MREPT technique shows that the recovered high-frequency electrical conductivity provides sufficient contrast to microstructural changes of tissues due to irradiation [14].

For the high-frequency conductivity, by assuming a local homogeneity, a direct algebraic inversion method has been introduced [7]. Although the algebraic inversion method is easy and recovers the conductivity values in each voxel, the assumption of local homogeneity produces artifacts around regions of complex conductivity structures even with noiseless data. There have been efforts to avoid assuming local homogeneity [6, 15, 16]. A phase-based conductivity imaging method including the conductivity gradient terms has been formulated by solving a convection-reaction partial differential equation [6]. The contrast-source inversion method applied to EPT (CSI-EPT) has been proposed to retrieve the conductivity and permittivity maps within a domain of interest even for strongly inhomogeneous tissue profiles [15, 17]. MREPT reconstruction methods have still suffered from interfering noise and undesired artifacts in the reconstructed conductivity map due to weak phase signals and defective regions.

Recently, electrical properties tomography based on deep learning approaches, have been introduced [18, 19]. However, deep learning techniques need exhaustive data sets in training to increase accuracy of reconstructed conductivity image. For directly matching from the B1 phase map to the high-frequency conductivity map, a typical deep learning technique is necessary to get data sets from a large number of human experiments. For these reasons, the proposed methods depend on realistic simulated data sets using the head models [18, 19].

In this paper, we propose a moving local window multilayer perceptron (MLW-MLP) neural network method to reconstruct the high-frequency conductivity, which is robust to the noise artifacts and prevents propagation artifacts due to defective regions. The high-frequency conductivity based on the electro-magnetic system is deeply related to the first and second differentiation of measured B1 transceive phase signals using an MRI scanner [20]. The proposed MLW-MLP uses the gradients and Laplacian of measured B1 phase data as the input layer in a local window consisting of neighboring voxels around a voxel in a region of interest (ROI). The input layer consists of the gradient and Laplacian of measured B1 phase data and the output of MLW-MLP is the conductivity values in the local window. We use rectifier linear unit (ReLU), batch normalization, *L*^2^-regularization, and mean absolute error (MAE) to summarize and assess the quality of the MLW-MLP machine learning model. After the training process, the output of MLW-MLP is the high-frequency conductivity distribution in the local window. By moving the local window with the stride 1, we recover the high-frequency conductivity image in ROI. Since the designed MLW-MLP recover the conductivity values in the local window, we determine a representative predicted conductivity value at a voxel included in the local window. To suppress the noise artifact and to increase the accuracy of predicted conductivity, a representative conductivity value is estimated as a weighted combination of conductivity values in each local window.

The proposed MLW-MLP method is proposed based on the following observations:

- The designed MLW-MLP included the measured phase map, *φ*, the gradients of *φ*, and the Laplacian of *φ* based on the physical electromagnetic system.
- The locally predicted conductivity image can avoid the deteriorated region affected by defective phase data by solving the direct global matrix system using the convection-diffusion equation ([6]).
- The multiple recovered conductivity images by moving a local window voxel-by-voxel can determine the noise level of conductivity at a voxel. It is possible to use the noise level at a voxel for suppress the noise artifacts without loss of the resolution of the conductivity.

To verify the proposed method, we generate the high-frequency conductivity using the convection-reaction partial differential equation with a small regularization parameter as the ground truth data [6]. The B1 phase signals are collected at 3 T MRI scanner from 8 subjects, using 5 subjects for training procedures and 3 subjects to predict the high-frequency conductivity distribution. The number of local windows for training datasets were 76992. To quantitatively verify the proposed deep learning method, we artificially destroyed the measured phase signals by adding random noise artifacts and generate defective regions. The accuracy and precision of the reconstructed conductivity distributions were evaluated, and the impact of different noise level and defect datasets were also investigated.

## Materials and methods

### High-frequency conductivity using B1-map

The high-frequency electrical tissue properties of conductivity *σ*_*H*_ and permittivity *ϵ*_*H*_ satisfy the following at Larmor frequency *ω*

$$\begin{array}{c} \nabla^{2}B_{1}=i\omega \mu_{0}\gamma_{H}B_{1}-\frac{\nabla \gamma_{H}}{\gamma_{H}}\times \left(\nabla \times B_{1}\right) \end{array}$$ (1)

where *γ*_*H*_ = *σ*_*H*_ + *iωϵ*_*H*_, **B**_1_ denotes the B1 field, and *μ*_0_ = 4*π* × 10^−7^ N/A^2^ is the magnetic permittivity of free space [7]. By assuming a local homogeneity (∇*γ*_*H*_ ≈ 0), the Eq (1) leads to a simple algebraic conventional MREPT algorithm:

$$\begin{array}{c} \gamma_{H}=\frac{\nabla^{2}B_{1}}{i\omega \mu_{0}B_{1}} \end{array}$$ (2)

For the positive (negative) rotating component of the transmit B1 field $B_{1}^{+}=|\;B_{1}^{+}\;|e^{i\varphi^{+}}$ ($B_{1}^{-}=|\;B_{1}^{-}\;|e^{i\varphi^{-}}$), by assuming *σ*_*H*_ ≫ *ωϵ*_*H*_, a phase-based convection reaction equation-based MREPT formula was derived as

$$\begin{array}{c} \nabla \varphi^{tr}\cdot \nabla \tau_{H}+\tau_{H}\nabla^{2}\varphi^{tr}-2\omega \mu_{0}=0 \end{array}$$ (3)

where *τ*_*H*_ denotes $\frac{1}{\sigma_{H}}$ and *φ*^*tr*^ = *φ*^+^ + *φ*^−^ is the measurable transceive phase using MRI [6].

To stabilize the formula (3), after adding an artificial diffusion term, the Eq (3) leads to

$$\begin{array}{c} -c\nabla^{2}\tau_{H}+\nabla \varphi^{tr}\cdot \nabla \tau_{H}+\tau_{H}\nabla^{2}\varphi^{tr}=2\omega \mu_{0} \end{array}$$ (4)

where *c* is a constant diffusion coefficient.

For the two dimensional case, a numerical differentiation of the Eq (4) for *τ*_*H*_ at a grid point (*x*_*i*_, *y*_*j*_) is written as

$$\begin{array}{c} \begin{array}{ccc} & & -c(\frac{\tau_{i+1,j}-2\tau_{i,j}+\tau_{i-1,j}}{{\left(\Delta x\right)}^{2}}+\frac{\tau_{i,j+1}-2\tau_{i,j}+\tau_{i,j-1}}{{\left(\Delta y\right)}^{2}})\\ & & +\frac{\tau_{i+1,j}-\tau_{i-1,j}}{2\Delta x}\cdot \frac{\partial \varphi^{tr}}{\partial x}+\frac{\tau_{i,j+1}-\tau_{i,j-1}}{2\Delta y}\cdot \frac{\partial \varphi^{tr}}{\partial y}\\ & & +\tau_{i,j}(\frac{\partial^{2}\varphi^{tr}}{\partial x^{2}}+\frac{\partial^{2}\varphi^{tr}}{\partial y^{2}})=2\omega \mu_{0} \end{array} \end{array}$$ (5)

Three dimension case is similar to the two dimension case in (5).

The discretized Eq (5) leads to the following global matrix system using the known boundary information:

$$\begin{array}{c} Ax=b \end{array}$$ (6)

where **A** is a staff matrix, **x** = (*τ*_1_, …, *τ_N_*)^*T*^, and **b** = (2*ωμ*_0_, …, 2*ωμ*_0_)^*T*^, respectively.

We note that the measured phase signal *φ*^+^ is continuous because its differentiations generate the edge information of the conductivity. The induced relations, (1) and (3), between the conductivity and the measured phase signal show that the contrast of conductivity is related to the differentiation of the phase signals. Measured phase signal *φ*^*tr*^ often includes local defects suffering from poor signal-to-noise-ratio (SNR) due to the low intensity of MR magnitude, especially when animals and humans are imaged. Since the global matrix system (6) includes twice differentiation of measured phase signal, the conductivity *σ*_*H*_ obtained by solving (6) propagates the severe noise artifact from the defective regions to the neighboring imaging area.

### Voxel-based moving local window deep learning for high-frequency conductivity

In MREPT, the B1 phase signals include the internal currents information induced by the secondary magnetic fields. The absolute conductivity value in a local homogeneous region and the edge variation of the conductivity are directly related to Laplacian and the gradient of measured phase signal *φ*, respectively. By taking into account the relationship between the conductivity and the measured B1 phase signals, the MLW-MLP model accepts multiple feature values consisting of the derivatives of B1 phase signals. To design voxel-based deep learning for high-frequency conductivity, a group of feature values in a local window surrounding a given voxel is used as input of MLW-MLP. The output of the MLW-MLP is the reconstructed conductivity values in the local window. Hidden layers use an activation function called rectified linear unit (ReLU), which simply truncates negative values.

At each **r** = (*x*, *y*), we will denote $F_{r}$ by the group of phases, *φ*, gradients, $\frac{\partial \varphi }{\partial x}$ and $\frac{\partial \varphi }{\partial y}$, and Laplacian values, ∇^2^*φ*, in a local window *w*_**r**_ = [*x* − *p*, *x* + *p*] × [*y* − *p*, *y* + *p*]. More precisely, $F_{r}=\left\{G_{1}^{{\text{w}}_{r}},G_{2}^{{\text{w}}_{r}},G_{3}^{{\text{w}}_{r}},G_{2}^{{\text{w}}_{r}}\right\}$, where $G_{1}^{{\text{w}}_{r}}=\left\{\varphi \left(s\right)\;|\;s\in {\text{w}}_{r}\right\}$, $G_{2}^{{\text{w}}_{r}}=\left\{\frac{\partial \varphi }{\partial x}\left(s\right)\;|\;s\in {\text{w}}_{r}\right\}$, $G_{3}^{{\text{w}}_{r}}=\left\{\frac{\partial \varphi }{\partial y}\left(s\right)\;|\;s\in {\text{w}}_{r}\right\}$, and $G_{4}^{{\text{w}}_{r}}=\left\{\nabla^{2}\varphi \left(s\right)\;|\;s\in {\text{w}}_{r}\right\}$. We represent MLW-MLP map as

$$\begin{array}{c} \sigma_{p}^{{\text{w}}_{r}}=\Phi F_{r} \end{array}$$ (7)

$\sigma_{p}^{{\text{w}}_{r}}$ denote the reconstructed conductivity values in the local window *w*_**r**_. To train MLW-MLP map *Φ*, we use the ground truth conductivity *σ*_*H*_ obtained by solving the discretized matrix system (6). We choose a small regularization parameter *c* in (4) to increase the accuracy of conductivity. To minimize the output errors on the feature set $F_{r}$, MLW-MLP consists of 4 hidden layers of nonlinearly-activating nodes. The number of neurons comprising the layer was 1024, 512, 256, and 128, respectively. The MLP network weights iteratively updates the thresholded ReLU. The window size of output layer was same to the size of moving local window of input layer. The designed method using the mixed data $F_{r}$ is processed by changing connection weights, based on the amount of error in the output compared to the expected conductivity values in the local window *w*_**r**_. The MLW-MLP is suitable for the proposed voxel-by-voxel recovery procedure and allows approximate solutions for the complex nonlinear conductivity recovery problem. Batch normalization (BN) was executed after each hidden layer to increase the stability of a neural network and improve the training accuracy. BN normalizes the output of a previous activation layer by subtracting the batch mean and dividing by the batch standard deviation within a batch of training images.

### Reconstruction of a representative conductivity value in a local window

The output of MLW-MLP predicts the conductivity values in the local window *w*_**r**_ corresponding to each voxel **r**. A natural way to determine a representative conductivity is to select the conductivity value at the center of local window:

$$\begin{array}{c} \sigma_{p}^{c}\left(r\right)≔\sigma_{p}^{{\text{w}}_{r}}\left(r\right) \end{array}$$ (8)

where **r** is the center voxe of *w*_**r**_.

To suppress the noise amplification, the conductivity value can be estimated as a weighted combination of conductivity values in the local window:

$$\begin{array}{c} \sigma_{p}^{\text{w}}\left(r\right)=\sum_{s\in {\text{w}}_{r}}w\left(r,s\right)\sigma_{p}^{{\text{w}}_{r}}\left(s\right) \end{array}$$ (9)

To determine an appropriate weighting factor *w*(**r**, **s**) such that *w*(**r**, **s**) ≥ 0 and $\sum_{s\in {\text{w}}_{r}}w\left(r,s\right)=1$, we shall define a non-spatial data-dependent distance, *D_N_*(**r**, **s**), and determine

$$\begin{array}{c} w\left(r,s\right)=\frac{1}{\zeta_{r}}e^{-D_{N}\left(r,s\right)}\;\text{for}\;s\in {\text{w}}_{r} \end{array}$$ (10)

where $\zeta_{r}=\sum_{s\in {\text{w}}_{r}}e^{-D_{N}\left(r,s\right)}$ is a normalization constant ensuring that $\sum_{s\in {\text{w}}_{r}}w\left(r,s\right)=1$.

We define a data-dependent distance *D_N_*(**r**, **s**) as

$$\begin{array}{c} D_{N}\left(r,s\right)≔\frac{\left|\;\sigma_{p}^{{\text{w}}_{r}}\left(r\right)-\sigma_{p}^{{\text{w}}_{r}}\left(s\right)\;\right|}{\eta (r)}, \end{array}$$ (11)

where *η*(**r**) is a denominator at the voxel **r**. Since the weighting factor *w*(**r**, **s**) depends only on the data-dependent distance *D_N_*(**r**, **s**), *w*(**r**, **s**) is calculated in the local window *w*_**r**_ consisting of neighboring voxels around the voxel **r**.

The parameter *η* quantifies how fast the weights decay depending on the similarity of respective patches. The filtering parameter *η* is selected by taking into account the noise variance of measured image. The predicted conductivity values at **r** are multiply determined due to the overlapped regions by moving the local windows. Let *S* = {**s** | **r** ∈ *w*_**s**_}. Then all predicted conductivity values in $g_{\sigma }^{\text{w}}\left(r\right)=\left\{\sigma_{p}^{{\text{w}}_{s}}\left(r\right)\;|\;s\in S\right\}$ are theoretically same without noise artifact. By estimating the noise standard deviation of the multiply determined conductivity values at **r**, we determine the denominator *η*(**r**) in (11) as

$$\begin{array}{c} \eta \left(r\right)\propto sd(g_{\sigma }^{\text{w}}\left(r\right)) \end{array}$$ (12)

where $sd(g_{\sigma }^{\text{w}}\left(r\right))$ is the standard deviation of $g_{\sigma }^{\text{w}}\left(r\right)$.

In summary, the contributions are two-fold:

1. Voxelwise MLW-MLP neural network at **r** with a family of groups: $F_{r}=\left\{G_{1}^{{\text{w}}_{r}},G_{2}^{{\text{w}}_{r}},G_{3}^{{\text{w}}_{r}},G_{4}^{{\text{w}}_{r}}\right\}$.
  - Prepare the family of gradient and Laplacian of B1 phase signals for the input layer: $\left(\varphi ,\frac{\partial \varphi }{\partial x},\frac{\partial \varphi }{\partial y},\nabla^{2}\varphi \right)$ in the local window *w*_**r**_.
  - Training MLW-MLP neural network: the input is $G_{1}^{{\text{w}}_{r}}=\left\{\varphi \left(s\right)\;|\;s\in {\text{w}}_{r}\right\}$, $G_{2}^{{\text{w}}_{r}}=\left\{\frac{\partial \varphi }{\partial x}\left(s\right)\;|\;s\in {\text{w}}_{r}\right\}$, $G_{3}^{{\text{w}}_{r}}=\left\{\frac{\partial \varphi }{\partial y}\left(s\right)\;|\;s\in {\text{w}}_{r}\right\}$, and $G_{4}^{{\text{w}}_{r}}=\left\{\nabla^{2}\varphi \left(s\right)\;|\;s\in {\text{w}}_{r}\right\}$ in the local window, the output of MLW-MLP is *σ*_*H*_ in the local window.
2. Determination a representative conductivity value in the local window.
  - Predict conductivity values in each local window using the trained model $\Phi F_{r}$.
  - Rearrange the overlapped conductivity values and estimate the noise level of conductivity value at **r**.
  - Determine the weighted averaged conductivity $\sigma_{p}^{\text{w}}\left(r\right)$ as in (9).

**Fig 1:**
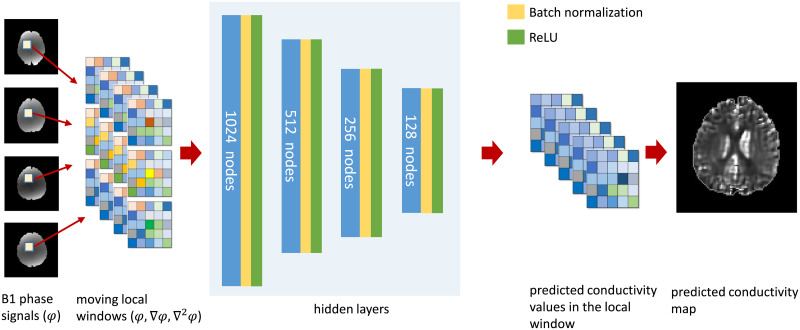
A graphical visualization of MLW-MLP neural network for high-frequency conductivity prediction.

Fig 1 shows a schematic of MLW-MLP neural network to predict the conductivity using the measured B1 phase signals.

### Experimental setup

MRI measurements were performed with eight healthy volunteers without a documented history of any disease were recruited. The participants were located inside the bore of a 3T MRI scanner with the head coil in transmit and a 32-channel RF head coil (Achieva TX, Philips Medical Systems, the Netherlands). All experimental protocols were approved by the institutional review board of Kyung Hee University (KHSIRB-16–033). All methods were carried out in accordance with the relevant guidelines and regulations and all participants provided written informed consent.

For MREPT imaging experiments, the multi-spin-echo pulse sequence with multiple refocusing pulses was adopted to minimize the measured noise. Before the data acquisition, we applied a volume shimming method with the volume defined to cover the brain region. Imaging parameters were as follows: repetition time *T*_*R*_ = 1500 ms, echo time *T*_*E*_ = 15 ms, number of echoes (NE) = 6, number of excitation (NEX) = 1, slice thickness = 4 mm, number of slices = 5, acquisition matrix = 128 × 128, field-of-view (FOV) = 240 × 240 mm^2^, and scan time = 16 min.

To reduce the noise artifacts, we used odd echoes of six measured complex MREPT signals to avoid the background phase signal due to the consecutive 180° RF pulses. Since the accumulated noise artifacts in the phase signal is inversely proportional to MR magnitude intensity, ${\tilde{S}}_{k},\;k=1,3,5$, the measured phase signal was optimized as a weighted averaging using the weight of [21]

$$\begin{array}{c} w_{k}=\frac{|{\tilde{S}}_{k}{|}^{2}}{|{\tilde{S}}_{1}{|}^{2}+|{\tilde{S}}_{2}{|}^{2}+{\left|{\tilde{S}}_{3}\right|}^{2}},\;k=1,3,5 \end{array}$$

## Results

We compared the predicted conductivity using MLW-MLP method to those by solving the convection-reaction partial differential equation in (4) with the diffusion term *c* = 0.02. The prepared data sets, including 76992 local windows with the five healthy volunteers, were trained with Adam which is an adaptive learning rate optimization algorithm for training deep neural networks [22]. Total epoch and batch size were 150 and 300, respectively. We designed 4 hidden layers, 1024, 512, 256, and 128 weights, batch normalization, and ReLU activation functions were applied. To stabilize the multilayer procedure, *L*^2^-regularization was applied to the MLP model. The used local window size was 9 × 9 and total training time was 3.25 min. This network was implemented in Keras and training was performed on a GPU (NVIDIA GeForce RTX 2070 super, 8GB RAM).

Performance of the proposed method was compared with the reconstructed conductivity image by solving PDE in (4) in terms of peak signal-to-noise ratio (PSNR) and relative *L*^2^-error:

$$\begin{array}{c} \text{PSNR}\left(X,Y\right)=10\log_{10}(\frac{M_{ax}^{2}}{MSE}) \end{array}$$ (13)

where $MSE=\frac{1}{mn}\sum_{i=1}^{m}\sum_{j=1}^{n}{\left(X\left(i,j\right)-Y\left(i,j\right)\right)}^{2}$ and *M*_*ax*_ is the maximum possible voxel value of the image of *X*. The relative *L*^2^-error is defined as

$$\begin{array}{c} L_{err}^{2}\left(X,Y\right)=\frac{{∥X-Y∥}_{2}}{{∥X∥}_{2}} \end{array}$$ (15)

**Fig 2:**
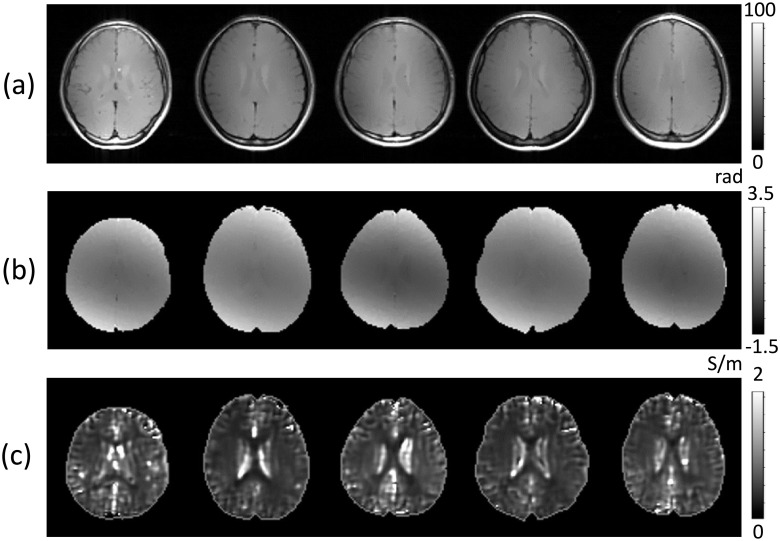
Training data sets with five healthy volunteers without a documented history of any disease. (a) MR magnitude images and (b) B1 phase images at the third imaging slice using the spin MR pulse sequence. (c) Reconstructed conductivity images by solving the convection-reaction partial differential equation in (4) with the diffusion term *c* = 0.02.

Fig 2 shows the prepared data sets for the inputs of MLW-MLP neural network: (a) and (b) are MR magnitude images and weighted B1 phase maps at the third imaging slice, respectively. Fig 2(c) shows the reconstructed conductivity distributions. To recover the conductivity map with the acquired transceiver phases of B1 maps (Fig 2(b)), we solved the convection-reaction partial differential equation (PDE) in (4) with the diffusion term *c* = 0.02. We used the reconstructed conductivity images in Fig 2(c) as the ground truth data sets for training procedures. The weights for five deep layers were adjusted to find patterns to make better predictions. We used the MLW-MLP model for predicting the conductivity map in the brain.

**Fig 3:**
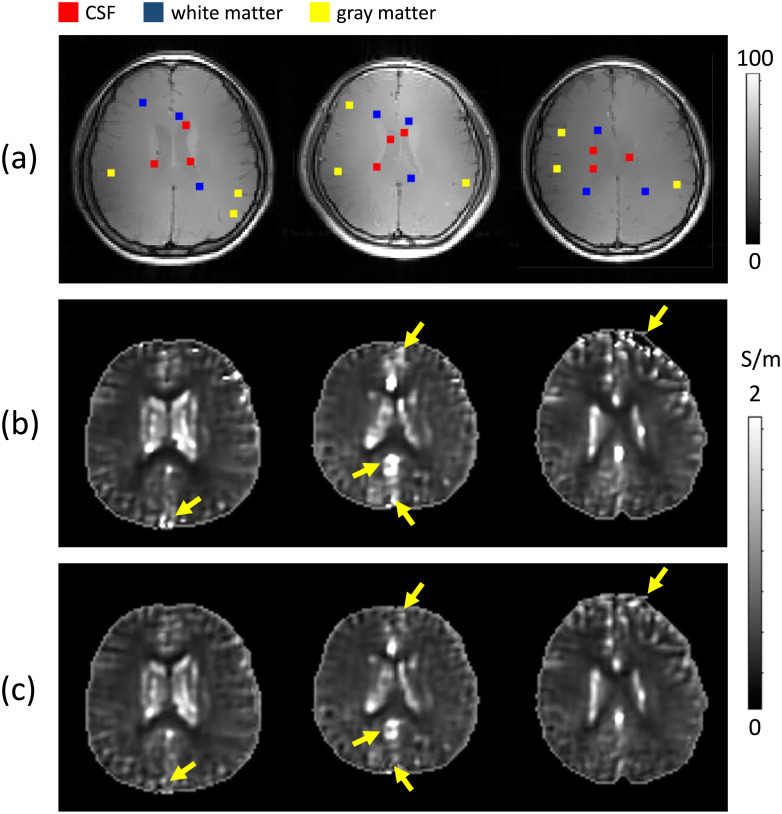
Test data sets with three healthy volunteers without a documented history of any disease and predicted conductivity images. (a) MR magnitude images. (b) Reconstructed conductivity images by solving the convection-reaction partial differential equation in (4) with the diffusion term *c* = 0.02. (c) Predicted conductivity image, $\sigma_{p}^{c}$, using MLW-MLP neural network.

Fig 3 shows the predicted results with three healthy volunteers data sets. Fig 3(a) shows the MR magnitude images at the third imaging slice. The reconstructed conductivity images by solving the convection-reaction PDE in (4) with the diffusion term *c* = 0.02 were displayed in Fig 3(b). Fig 3(c) shows the predicted conductivity images using the predicted conductivity value, $\sigma_{p}^{c}$, at the center of each local window.

To stabilize the training procedure, we used the *L*^2^-regularization, 0.01, as the kernel regularizer to apply penalties on layer parameters. Since the proposed method reconstructs the conductivity depending on the local characteristics of the measured B1 phase signal, compared to the reconstruction results using the global system that affects the local defects in the surrounding regions, the imaging quality of the predicted conductivity was improved in the local regions (designated by the yellow arrows in Fig 3).

Table 1 shows the estimated conductivity values in CSF, gray matter, and white matter regions (CSF: red spots, white matter: blue spots, gray matter: yellow spots in Fig 3(a)). The known reference values of high-frequency conductivity are 1.65∼1.92 (CSF), 0.59∼0.63 (gray matter) and 0.30∼0.43 S/m (white matter) at 128 MHz [23–25]. The predicted conductivity values, $\sigma_{p}^{c}$, in CSF regions were slightly lower than *σ*_*H*_, while the conductivity values of $\sigma_{p}^{c}$ in white matter regions were higher than *σ*_*H*_ due to the adopted *L*^2^-regularization parameter to prevent overfitting in the deep learning process.

**Table 1:** Estimated conductivity values, *σ*_*H*_, by solving the global matrix system in (4). Predicted conductivity values, $\sigma_{p}^{c}$, at the center of local window.

		*σ*_*H*_	$\sigma_{p}^{c}$
case1	CSF	1.73±0.37	1.43±0.35
	gray matter	0.63±0.07	0.66±0.17
	white matter	0.24±0.04	0.28±0.06
case2	CSF	1.57±0.15	1.26±0.13
	gray matter	0.69±0.11	0.65±0.14
	white matter	0.31±0.04	0.32±0.06
case3	CSF	1.82±0.25	1.62±0.24
	gray matter	0.58±0.07	0.61±0.05
	white matter	0.30±0.02	0.33±0.03

Table 2 show the evaluated PSNR values and relative *L*^2^-errors between the recovered conductivity image, *σ*_*H*_, by solving the elliptic partial differential equation in (4) and the predicted conductivity images $\sigma_{p}^{c}$.

**Table 2:** Estimated PSNR and relative *L*^2^-errors for the three test data sets.

	case1	case2	case3
$\text{PSNR}(\sigma_{H},\sigma_{p}^{c})$	13.02	12.42	10.62
$L_{err}^{2}\left(\sigma_{H},\sigma_{p}^{c}\right)$	0.17	0.17	0.22

**Fig 4:**
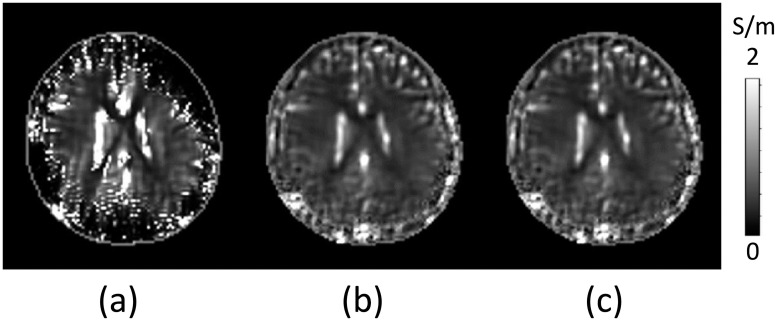
Predicted conductivity images using defective data. (a) Reconstructed conductivity images using the defective B1 phase signals. The reconstructed conductivity image including defective regions by solving the PDE in (4) with the diffusion term *c* = 0.005. (b) Predicted conductivity image, $\sigma_{p}^{c}$, using MLW-MLP neural network. (c) Predicted weighted combination, $\sigma_{p}^{\text{w}}$, of conductivity values in each local window.

To verify the proposed deep learning method, we tested two cases. We artificially destroyed the measured phase signals by adding random noise artifacts and generate defective regions. To investigate the effect of defective regions, we included the whole head region, where the conductivity reconstruction often failed in scalp and skull regions due to the boundary artifacts and low conductivity values. Fig 4(a) shows the reconstructed conductivity map in the whole head region by solving the global matrix system induced from the PDE in (4) with the diffusion parameter *c* = 0.005. Since the noise characteristics are different at each region due to the magnitude intensity, tissue properties, motion, imaging parameters, etc, it is difficult to determine an appropriate diffusion parameter *c* in (4). Fig 4(a) shows the propagated noise artifact from the defective regions to the surrounded region by solving the global matrix system in (6). Since the proposed voxel-based deep learning method included sufficient training data sets, conductivity maps can be locally predicted regardless of the condition of the boundary and partially severe noise artifacts. The predicted conductivity maps using the trained NLW-MLP model were displayed in Fig 4(b) and 4(c), respectively. Fig 4(b) shows the predicted conductivity map, $\sigma_{p}^{c}$, using the predicted conductivity value at the center of each local window. By taking a weighted combination of predicted conductivity values in the local window, Fig 4(c) shows the weighted conductivity images $\sigma_{p}^{\text{w}}$.

**Fig 5:**
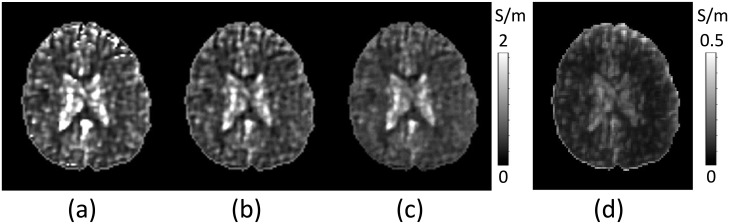
Predicted conductivity images using noisy B1 data. (a) Reconstructed conductivity images using the noisy B1 phase signals. The measured B1 phase map was artificially destroyed by adding Gaussian random noise. (b) Predicted conductivity image, $\sigma_{p}^{c}$, using MLW-MLP neural network. (c) Predicted weighted combination, $\sigma_{p}^{\text{w}}$, of conductivity values in each local window. (d) The noise standard deviation of the multiply determined conductivity values at **r**.

In Fig 5, the measured B1 phase signals were artificially destroyed by adding Gaussian random noise. Using the noise added B1 phase signals, Fig 5(a) shows the reconstructed conductivity map, *σ*_*H*_, in the brain region by solving the global matrix system in (6) induced from the PDE in (4) with the diffusion parameter *c* = 0.02. The predicted conductivity maps, $\sigma_{p}^{c}$ at the center of each local window, and $\sigma_{p}^{\text{w}}$ using the predicted conductivity values in each local window were displayed in Fig 5(b) and 5(c), respectively. To calculate a weighted combination, $\sigma_{p}^{\text{w}}$, of predicted conductivity values in the local window, we determine the denominator *η*(**r**) in (11) using the noise standard deviation of the multiply determined conductivity values at **r**. Fig 5(d) shows the standard deviation of $g_{\sigma }^{\text{w}}\left(r\right)=\left\{\sigma_{p}^{{\text{w}}_{s}}\left(r\right)\;|\;s\in S\right\}$.

We compared *σ*_*H*_, $\sigma_{p}^{c}$ and $\sigma_{p}^{\text{w}}$ to the reconstructed conductivity $\sigma_{H}^{t}$ corresponding to the noiseless B1 signals, which were obtained by solving the global matrix system in (6). The evaluated PSNR values and relative *L*^2^-errors are given in Table 3. As expected, the image quality of $\sigma_{p}^{\text{w}}$ was better than the others.

**Table 3:** Estimated PSNR and relative *L*^2^-errors for the noisy data shown in Fig 5.

	*σ*_*H*_	$\sigma_{p}^{c}$	$\sigma_{p}^{w}$
PSNR	8.69	9.60	10.11
$L_{err}^{2}$	0.40	0.33	0.29

We compared the proposed MLW-MLP method with two methods using only B1 phase data. The first method, MLW-MLP(phase), was to use only B1 phase data in our proposed MLW-MLP method. Except for the sliding window size, the depth and parameters of the network were the same as the proposed MLW-MLP method.

Another method, MLW-CNN(phase), used 2D convolutional neural networks (CNNs) instead of multi-layer perceptrons. The network architecture was a 5 layer CNNs. For the first four layers, 128, 64, 64 and 32 filters of size 3 × 3 are used, and batch normalization was added between convolution and ReLU. For the last layer, 1 filter of size 3 × 3 was used to reconstruct the output. Total epoch and batch size were 150 and 300, respectively.

Tables 4 and 5 show the evaluated PSNR values and relative *L*^2^-errors between *σ*_*H*_ and the predicted conductivity images $\sigma_{p}^{c}$, respectively, as the window size changes. It can be seen that the effective window size used in MWL-MLP(phase) is 9 × 9. MLW-CNN(phase) can achieve better PSNR and relative *L*^2^-errors as the window size increases, but the calculation time increases. Our proposed MWL-MLP with the window size 9 × 9 can achieve the best PSNR results and relative *L*^2^-errors than the other methods.

**Table 4:** Estimated $\text{PSNR}(\sigma_{H},\sigma_{p}^{c}o$ results for different methods with multiple window sizes.

	MLW-MLP(phase)					MLW-CNN(phase)				
window size	5×5	9×9	13×13	17×17	21×21	13×13	17×17	21×21	25×25	29×29
case1	10.83	12.07	11.95	11.35	10.78	11.00	10.96	11.02	10.74	11.05
case2	10.54	12.06	11.93	11.07	10.38	11.31	11.47	11.66	12.00	11.96
case3	9.15	10.42	10.23	9.13	8.66	9.64	9.61	9.68	10.01	10.02

**Table 5:** Estimated $L^{2}\left(\sigma_{H},\sigma_{p}^{c}\right)$ results for different methods with multiple window sizes.

	MLW-MLP(phase)					MLW-CNN(phase)				
window size	5×5	9×9	13×13	17×17	21×21	13×13	17×17	21×21	25×25	29×29
case1	0.27	0.21	0.21	0.24	0.28	0.26	0.27	0.26	0.28	0.26
case2	0.26	0.18	0.19	0.23	0.27	0.22	0.21	0.20	0.19	0.19
case3	0.31	0.23	0.24	0.32	0.35	0.28	0.28	0.28	0.26	0.26

## Discussion

Predicting the conductivity from measured B1 phase map needs careful understanding on the relationship between the B1 phase signal and conductivity distribution to be imaged. Based on the electromagnetic system, the noiseless B1 image is continuous and piece-wise smooth. Since the measured B1 phase map has no conventional edge information, it is difficult to design a deep learning model directly from the B1 phase map (input) to the conductivity distribution (output). In this paper, we focused on the relationship between the differentiation of B1 phase map and the conductivity map based on Maxwell equation. In this paper, for training the MLW-MLP deep learning, the recovered conductivity using the partial differential equation based on the Maxwell equation is used as the ground truth data. We used the regularization parameter *c* of the convection reaction Eq (4) as small as possible. The reconstruction method using the convection reaction equation has the advantage of being able to reconstruct the conductivity without boundary artifact, but the diffusion term including the regularization parameter *c* can disturb the absolute conductivity values and propagate severe artifacts from defective regions to the enclosed regions. The developed reconstruction methods for high-frequency conductivity using measured magnetic flux density is still insufficient to consider to be considered the ground truth. The predicted conductivity values in the gray matter, white matter, and CSF regions were slightly lower than the known reference conductivity values, respectively. One reason is to use the ground truth conductivity depending on the regularization parameter *c* of the convection reaction Eq (4) and the other reason relates to the designed MLW-MLP model which includes *L*^2^-regularization to stabilize the learning process.

The proposed method reconstructs the contrast of conductivity voxel-by-voxel, which blocks the propagation of severe accumulated noise from the defective region to the ROI. We used 9×9 local window size to reflect the surrounded pattern at a voxel. Although we have no exact formula to choose the optimal size of sliding window, the proposed MLW-MLP using a local differential characteristic of B1 phase data shows the best performance with the window size 9×9. The determined window size includes the surrounded voxels for computing the first and second order numerical derivatives. On the other hand, CNN’s method increases training time as the number of windows increases, although the error decreases slightly.

Since the absolute value and the edge information of conductivity are closely related to the differential of measured B1 phase signal, the accurate conductivity recovery using the relationship between conductivity and measured B1 phase signal mainly depends on whether the local characteristics of the B1 phase signal are well included in a machine learning scheme. By these observations, the proposed MLW-MLP successfully predicted the conductivity, and we compared the proposed method with the conventional CNN. There are many deep learning methods including CNN, recurrent neural network (RNN), denoising autoencoder (DAE), and long short-term memory (LSTM). Considering the physical properties of measurable magnetic flux density and electrical conductivity, we plan to design a better clinically applicable deep learning method.

Electrical brain stimulation (EBS) techniques, such as transcranial direct current stimulation (tDCS) and deep brain stimulation (DBS), are promising treatments for human disorders ([26–30]). Since there is no clear explanation for the mechanism, EBS studies have relied on computational modeling using reference conductivity values in the whole brain region. To solve this problem, the proposed MLW-MLP deep learning method can reconstruct the whole brain conductivity map by avoiding defect areas for visualizing internal current density and electric field caused by the EBS.

## Conclusion

We have proposed MLW-MLP neural network that is capable of visualizing the high-frequency conductivity map in the human brain from measured B1 phase signals using a conventional 3 T MRI scanner with significantly improved conductivity image quality. By taking into account the relationship between the conductivity and the measured B1 phase signals, the MLW-MLP model accepts multiple inputs consisting of Laplacian and the gradient of measured phase signal. The MLW-MLP method quickly and stably determine the high-frequency conductivity using a trained model without solving the complex global matrix system. Conventional MREPT techniques are difficult to apply to various organs in the human body, including bones, fat, and muscles, because the quality of B1 phase map is very poor in some defect regions. Since the MLW-MLP model locally recovers the conductivity values by observing the local characteristics of B1 phase signals, it can be applicable to other organs while avoiding the artifact propagation due to defective data. The experimental results demonstrate the effectiveness of MLW-MLP neural network suppresses noise artifacts and predicts the conductivity without being affected by severe noise in defect regions.

## Supporting information

S1 File (MAT)

S2 File (MAT)

S3 File (MAT)

S4 File (MAT)
